# The Influence of Temporary Epiphysiodesis of the Proximal End of the Tibia on the Shape of the Knee Joint in Children Treated for Leg Length Discrepancy

**DOI:** 10.3390/jcm13051458

**Published:** 2024-03-02

**Authors:** Grzegorz Starobrat, Anna Danielewicz, Tomasz Szponder, Magdalena Wójciak, Ireneusz Sowa, Monika Różańska-Boczula, Michał Latalski

**Affiliations:** 1 Department of Paediatric Orthopaedics, Medical University of Lublin, 20-093 Lublin, Poland; starobrat@o2.pl (G.S.); michallatalski@umlub.pl (M.L.); 2 Department and Clinic of Animal Surgery, Faculty of Veterinary Medicine, University of Life Sciences, 20-612 Lublin, Poland; tomasz.szponder@up.lublin.pl; 3 Department of Analytical Chemistry, Medical University of Lublin, 20-093 Lublin, Poland; magdalena.wojciak@umlub.pl (M.W.); ireneusz.sowa@umlub.pl (I.S.); 4 Department of Applied Mathematics and Computer Science, University of Life Sciences in Lublin, 20-033 Lublin, Poland; monika.boczula@up.lublin.pl

**Keywords:** leg length discrepancy, temporary epiphysiodesis, eight-plate, growth plate, knee deformity

## Abstract

**Background:** Leg length discrepancy (LLD) is a common problem in the daily clinical practice of pediatric orthopedists. Surgical treatment using LLD temporary epiphysiodesis with eight-plate implants is a minimally invasive, safe, and well-tolerated procedure that provides good treatment effects with a relatively low percentage of complications. The main aim of this retrospective study was to determine the effect of epiphysiodesis on the shape of the proximal tibia. **Methods:** The retrospective study was based on medical records from 2010 to 2019. Radiographs taken before the epiphysiodesis and at 6-month intervals until the end of the treatment were investigated. A total of 60 patients treated for LLD were included in the study (24 girls, 36 boys). They were divided into three groups depending on the duration of the LLD treatment: group I (18 months), group II (30 months), and group III (42 months of treatment). Radiological parameters were assessed, including the roof angle (D), the slope angles (α and β), and the specific parameters of the tibial epiphysis, namely LTH (lateral tubercle height), MTH (medial tubercle height), and TW (tibial width). **Results:** The roof angle decreased in all the groups, which was accompanied by an increase in the β or α angle. LTH, MTH and TW also increased, and the differences before and after the treatment for the treated legs were statistically significant in all the studied groups. The greatest change in the shape of the articular surface of the proximal tibia occurred after 42 months of treatment. **Conclusions:** The study showed that epiphysiodesis affects the proximal tibial articular surface over prolonged treatment. Thus, there is a need for future long-term follow-up studies to elucidate the potential effects of LLD egalization.

## 1. Introduction

Leg length discrepancy (LLD) of the lower limbs may result from structural deformations, i.e., real differences in the length of bones that make up a given limb segment [1,2,3]. Leg length discrepancy occurs in approximately two-thirds of the population of children. LLD up to approximately 5 mm occurs in approximately 10–12% of the population, and LLD above 10 mm occurs in approximately 4% of the population. 

The modern Multiplier app and Paley Growth app include a number of features used in a variety of clinical contexts, including upper- and lower-limb LLD calculation, epiphysiodesis time, and limb growth charts. It also contains information on other growth disorders [4].

Epiphysiodesis is a method of treating LLD that involves surgically blocking the growth plate in order to inhibit the growth of the longer limb. The first procedures involved irreversible destruction of the bone growth region, and the following years brought the development of reversible methods enabling safer control of limb growth [5].

**Figure 1:**
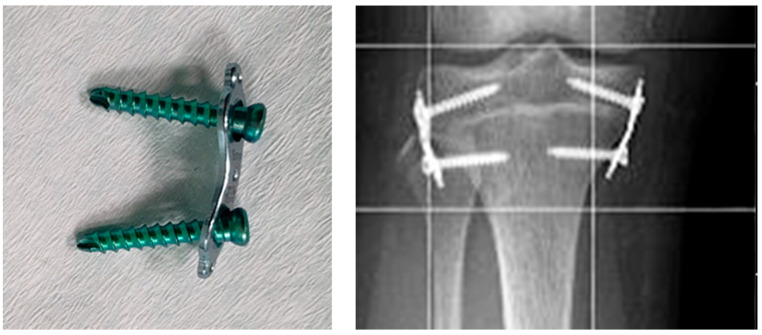
Eight-plate implant and X-ray image of the knee joint showing eight-plate implants used for the treatment of leg length discrepancy.

Epiphysiodesis with flexible plates in the shape of the figure “8” and two screws (Figure 1) was introduced in 2007 by Stevens (Orthofix; McKinney, TX, USA).

These implants were developed as a response to complications that occurred when using Blount staples. The strong compression with a stiff pin damaged the growth plate, which prevented it from functioning after the implant was removed. Other complications included damage or dislodgement during treatment [6].

There are no studies in the literature presenting the influence of temporal epiphysiodesis on the shape of the knee articular surface. Most of the available works on epiphysiodesis analyze the degree of its effectiveness—compensation of LLD—in various contexts. The impact of epiphysiodesis on the shape of the knee joint, the duration of treatment followed by joint deformation, and, consequently, biomechanics disorders has not been subjected to a broader analysis so far. Thus, the main aim of this study was to determine the effect of epiphysiodesis on the shape of the proximal tibia and establish a time frame for the safe use of this procedure. The following radiological parameters were assessed: the roof angle (D), the slope angles (α and β), and the specific parameters of the tibial epiphysis, namely LTH (lateral tubercle height), MTH (medial tubercle height), and TW (tibial width).

## 2. Materials and Methods

### 2.1. Selection of Participants

The study protocol and consent form were approved by the Bioethics Committee of the Medical University of Lublin (number KE-0254/81/2020, dated 30 April 2020). Our study was retrospective in nature. Medical records from 2010 to 2019, available in the archives of the Pediatric Orthopedic Clinic at the Medical University of Lublin, were analyzed. The inclusion criterion was idiopathic leg length discrepancy of lower limbs treated by temporary epiphysiodesis of the proximal tibia using eight-plate implants. The indication for the treatment was the presence of an active growth plate, indicating skeletal immaturity. The exclusion criteria included an etiology of shortening other than idiopathic and surgical procedures in the limb segment planned for treatment.

In the end, 60 cases were qualified for the study (24 girls, 36 boys). They were divided into 3 groups depending on the duration of the LLD treatment (group I (18 months), group II (30 months), and group III (42 months)). The characteristics of the groups are given in Table 1.

### 2.2. Study Design

**Figure 2:**
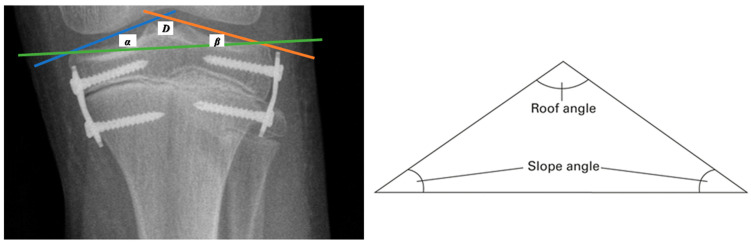
A graphic image of the tibial articular line (green line), the medial line of the tibial plate inclination (blue line), and the lateral tibial plate slope (orange line), as well as a graphical representation of the roof angle.

Radiographs taken before the planned epiphysiodesis procedure and at intervals of +/− 6 months until the end of the treatment were assessed. A full-length standing AP radiograph of the lower limbs (body X-ray) was used to assess angular and linear parameters. A single radiographic exposure of both lower limbs was performed, with the radiation beam centered on the knees from a distance of approximately 180 cm and the patient standing upright with both patellas pointing directly forward. To illustrate changes in the tibial plate surface expressed in degrees, measurements of the angles of the triangle proposed by R. Sinha from the connection of the tibial articular line (the line connecting the external highest points of the tibial condyles—green line in Figure 2), the medial line of the tibial plate inclination (the line between the highest point in the projection of the medial intercondylar tubercle and the point defining the outermost upper end of the medial condyle—blue line in Figure 2) (angle α), and the lateral line of the tibial plate inclination (the line between the highest point in the projection of the lateral intercondylar tubercle and the point defining the outer highest upper end of the lateral condyle—orange line in Figure 2) (angle β) were performed [7].

The “tibial roof” angle (D) is included between the medial and lateral lines of the tibial plate inclination, defined as the result of the difference in the acute angles of the triangle formed by the lines (the medial tibial plate inclination line, the lateral line of the tibial plate inclination, and the tibial articular line (180 minus α, minus β)) [7].

**Figure 3:**
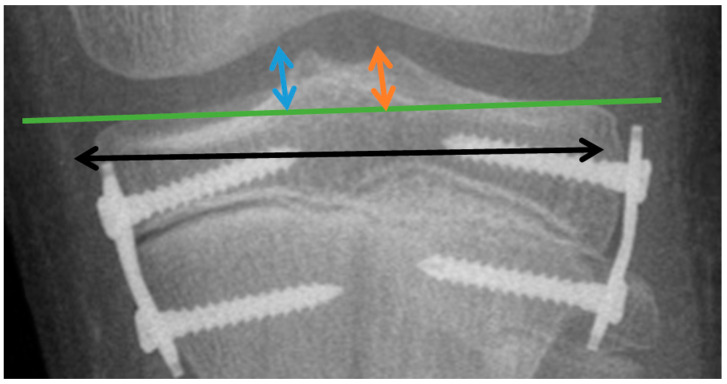
Graphical display of linear parameters. MLH (dimension measured from the tibial articular line to the highest point in the projection of the medial intercondylar tubercle) (blue arrow); LTH (dimension measured from the tibial articular line to the highest point in the projection of the lateral intercondylar tubercle) (orange arrow); and TW (width of the tibial epiphysis measured between the widest points of the proximal tibial epiphysis, one located on the lateral and the other on the medial cortex) (black arrow).

To illustrate changes in the tibial plate surface expressed in millimeters, the changes in the height of the intercondylar tubercles of the medial MLH (dimension measured from the tibial articular line to the highest point in the projection of the medial intercondylar tubercle; blue arrow) (Figure 3) and lateral LTH (dimension measured from the tibial articular line to the highest point in the projection of the lateral intercondylar tubercle; orange arrow) (Figure 3) were assessed. Changes in the width of the distal tibial epiphysis were illustrated by the TW parameter (width of the tibial epiphysis measured between the widest points of the proximal tibial epiphysis, one located on the lateral and the other on the medial cortex; black arrow) (Figure 3).

Data regarding the following characteristics (parameters) were collected for each person. For each feature, 4 measurements (group I), 6 measurements (group II), and 8 measurements (group III) were performed and repeated at equal time intervals (every 6 months). Observations were made for both the longer (treated) limb (“d”) and the shorter (non-treated) limb (“k”). 

### 2.3. Statistical Analysis

Each measurement was performed three times. Statistical evaluation of the data was performed using Statistica ver. 13.1 software. The significance level of the tests was α = 0.05. Since no normal distribution of the characteristics (Shapiro–Wilk test, *p* < 0.05) and no homogeneity of the relevant variances (Levene’s test) were confirmed, non-parametric tests were used to assess the relationships. When comparing measurements between subsequent time points, the Friedman test (equivalent to one-way analysis of variance for repeated measurements) was used, and the Wilcoxon paired test was used to compare two dependent features. Box-and-whisker plots were made to illustrate the changes.

## 3. Results

In this study, changes in the tibial plate surface were assessed using measurements of the D, α, and β angles (degree value), as well as the parameters of the tibial epiphysis (in mm), both after the treatment and at various time periods.

A decrease in the D angle reflects the elevation of both tubercles of the tibial plate, an increase in the α angle illustrates the lowering of the medial part of the tibial plate, and an increase in the β angle reflects the lifting of the lateral part of the tibial plate. In turn, an increase in LTH and MTH indicates a change in the shape of the articular surface of the tibia to a more conical one due to the apparent hyperactivity of the middle part of the growth plate. This may result in the loosening of the ACL (anterior cruciate ligament) and the PCL (posterior cruciate ligament), consequently creating a sense of instability.

### 3.1. Changes in the Investigated Parameters Evaluated after the Completion of the Treatment

The results for all the investigated parameters expressed as the difference between the values obtained after and before the treatment and the statistical analysis of the significance of the differences between the values obtained at the beginning and after the treatment are summarized in Table 2 and Table 3, respectively.

In groups I, II, and III, the D angle decreased significantly during the treatment, which was accompanied by an increase in the β or α angle. The lateral tubercle (LT) and medial tubercle (MT) height and the tibial width (TW) increased, and the differences before and after the treatment for the treated legs were statistically significant in all the studied groups. TW also changed in a statistically significant manner in the non-treated legs. In turn, the lateral tubercle height in the non-treated legs only changed in the boys in groups II and III and in the girls in group II. No statistically significant differences were observed for MT in the boys. 

### 3.2. Changes in the Investigated Parameters Evaluated Every Six Months during the Treatment

To track changes in the tibial plate surface over time, the parameters were assessed every six months. The results were expressed as the difference in measurements for the treated limb (longer—d) and the non-treated limb (shorter—k). The goal of this part was to verify whether the changes in parameter values at adjacent time points were the same for the non-treated limb (k) and the treated limb (d). For this purpose, a non-parametric Wilcoxon test was performed. The results for groups I, II, and III are shown in Table 4, Table 5 and Table 6, respectively. 

Statistically significant differences in parameter D were demonstrated in all the groups (*p* < 0.05), but in group I of the boys, only in the period of 12–18 months (Table 4). In group II, statistically significant differences were observed in the period of 6–18 months (boys) and 6–24 months (girls) (Table 5). In group III, the changes were observed from 6 to 18 and from 24 to 30 months in the group of girls, and from 6 to 18 and from 30 to 42 months in the boys (Table 6). The α angle increased significantly during the treatment. In group I, changes occurred from 0 to 18 months (girls) and from 0 to 12 months (boys) (Table 4). In group II, visible changes were observed in the periods of 12–30 months (girls) and 0–12 and 24–30 months (boys) (Table 5). Statistically significant differences in the girls from group III were observed between 6 and 24 months, and differences in the group of boys were notable in the last stage of treatment, i.e., at 42–48 months (Table 6). The highest changes in the β angle were observed in the girls from group III (Table 6) and in the boys from group II (Table 5), where this parameter increased significantly during most of the treatment period. The highest differences in the lateral tubercle height and in the medial tubercle height in the different treatment periods were found in the groups of girls and in the boys from groups I and II (Table 4 and Table 5, respectively). Statistically significant changes for TW were noted for the girls in groups II and III and for the boys in group II in almost all the investigated period ranges (Table 5 and Table 6).

**Figure 4:**
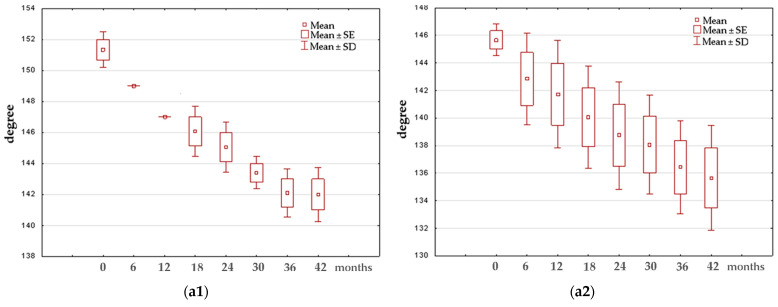

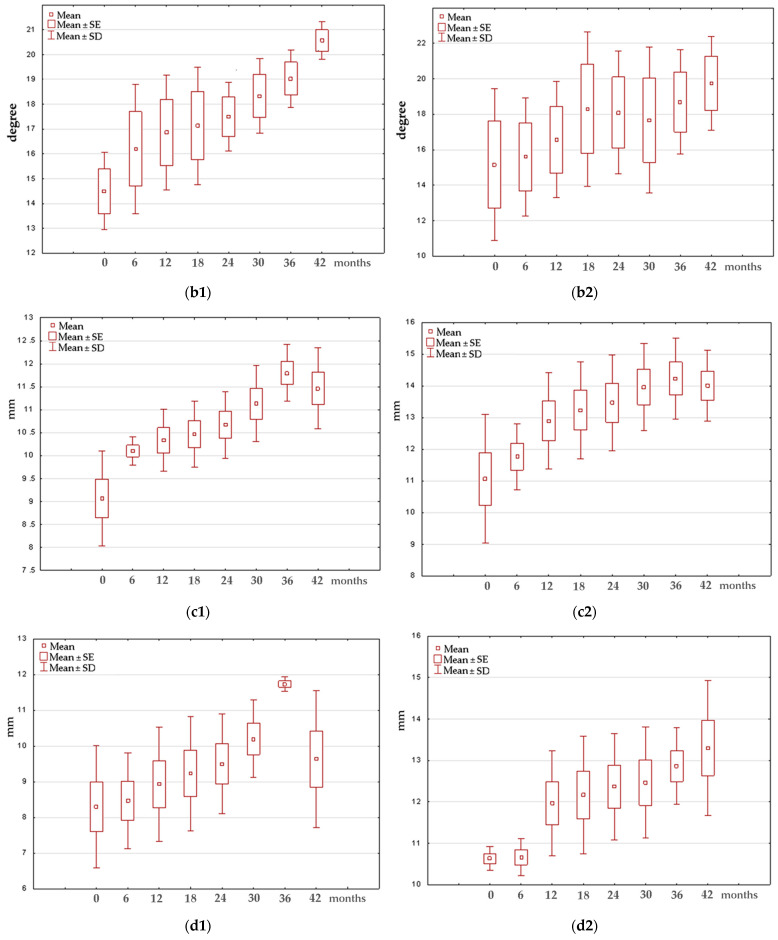

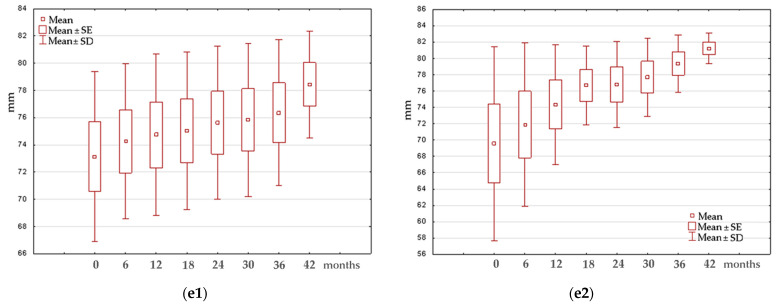
Example of box-and-whisker plots illustrating changes in the investigated parameters over the specific time periods for group III: (**a1**,**a2**)—roof angle (D) in the group of girls and boys, respectively; (**b1**,**b2**)—slope angle β in the group of girls and boys, respectively; (**c1**,**c2**)—lateral tubercle height (LTH) in the group of girls and boys, respectively; (**d1**,**d2**)—medial tubercle height (MTH) in the group of girls and boys, respectively; (**e1**,**e2**)—tibial width (TW) in the group of girls and boys, respectively.

Box-and-whisker plots were created to illustrate the changes noted in the specific time periods. An example presenting the results obtained for group III is shown in Figure 4.

## 4. Discussion

Epiphysiodesis using eight-plate implants is a minimally invasive method of correcting LLD. It is well tolerated and accepted by pediatric patients and their parents. It is offered to patients as an alternative to long surgical treatment requiring external fixators and burdened with a higher risk of complications, e.g., lengthening using the Ilizarov method. We have found several scientific papers in which, in addition to the effects of LLD treatment with temporary epiphysiodesis-egalization, complications are also described. These are mainly cases of implant damage—its rupture, dislodgement, or displacement. They also describe permanent damage to the growth plate; after the implant was removed, the plate did not resume its function, causing a disruption of the leg axis [8,9,10,11,12,13,14,15]. Due to the limited literature on the analysis of changes in the shape of the proximal tibial base treated with an eight-plate implant, in this discussion, we used reports of authors examining the results of LLD treatment also using other types of implants.

In 2007, P. Stevens began research on an implant intended to reduce the number of complications often observed during epiphysiodesis treatment of LLD using a Blount staple. In the same year, the researcher published an article in which he described the technique of asymmetrical temporary epiphysiodesis using a flexible plate in the shape of the figure “8” and two screws (Orthofix GmbH, Lewisville, TX, USA) [6]. This method was originally dedicated to the treatment of leg axis disorders [13,14,16,17]. The simple surgical technique of reducing the number of implants to one (in the case of staples, several were used) and the good results presented by other authors resulted in the extension of the indications, and this method was used for symmetrical epiphysiodesis in the treatment of LLD [18,19]. The introduction of these implants solved the main problem of temporary epiphysiodesis, i.e., permanent damage to the growth plate. The implant, which facilitates gradual inhibition of growth, does not damage the growth plate. The screws are not rigidly mounted in the plate, and the angle can be increased (up to a maximum of 60 degrees). Further mechanisms of gradual epiphysiodesis include the narrowing of the plate by half of its length and the flexibility of the material from which it is made. The plate begins to bend under the pressure of growth forces. This implant design also moves the point of support of the epiphysiodesis forces away by approximately 30 mm from its outer part, which increases the surface area of impact of these forces [6]. We had similar observations when analyzing the study groups. We did not observe any cases of permanent growth damage. Patients whose leg normalization occurred before the end of growth had their implants removed, and follow-up examinations confirmed further normal growth of the operated limb. We did not observe any damage to the implant itself, but deformations of the plate were visible, especially in the patients from group III.

According to the theory of the inventors of the implant, the use of eight-plate implants in symmetric epiphysiodesis causes different effects on the growth plate, depending on the distance from the implant. Columns of chondrocytes located closer to the implant are inhibited more strongly, and those closer to the center of the joint are less inhibited. This provides a graphic image of the triangle formed by the medial line of the tibial plate slope, the lateral line of the tibial plate slope, and the tibial articular line. In turn, the angle defined as the result of the difference between the acute angles of the triangle, i.e., the roof angle D, was described by R. Sinha et al. in 2018 as the main parameter determining possible disorders of the articular surface of the tibia [7]. This is the first work that, almost 10 years after the introduction of eight-plate implants for the treatment of LLD, describes changes in the articular surfaces of the knee. In his work, the researcher tried to graphically and mathematically present how the shape of the tibial epiphysis changes during LLD treatment with epiphysiodesis using eight-plate implants. In the material of 42 patients, the author proved that the shape of the joint changed in almost half of the examined patients (46%), and the roof angle decreased by an average of 5 degrees (from 1 to 18 degrees) during the treatment. Sinha’s work, however, had limitations, namely, the size of the group of patients treated due to LLD (only 8 cases) and their combination with the group of patients treated due to KD axis disorder (34 cases). The average follow-up time was 1.8 years (from 0.5 to 5 years).

The results presented by R. Sinha confirm the observations reported by scientists who compared the results of temporary epiphysiodesis with the final one. They claim that temporary epiphysiodesis with eight-plate implants causes a change in the shape of the joint. This may be related to the design of the implant, i.e., the possibility of changing the angle of the screw in relation to the plate causes a delay in the process of epiphysiodesis [18,20].

In 2016, E. Gaumetou et al. assessed the effectiveness of LLD treatment using eight-plate implants on a group of 32 patients. The results were very unsatisfactory; in the case of the proximal tibial metaphysis, only 13 patients (42%) achieved the expected KD compensation. Eight patients (20%) reported pain up to 18 months after treatment, and five (12.5%) required revision of the implant position. The author also reported asymmetries in the shape of the tibial plate of the treated limb visible on radiographs [18].

In turn, in 2019, Borbasa presented the results of a comparison of irreversible epiphysiodesis with temporary epiphysiodesis (using eight-plate implants). The researcher compared the results of KD correction, which spoke in favor of irreversible epiphysiodesis (group of 21 patients). After a year, a reduction in LLD by an average of 8.4 mm was achieved in this group compared to the reversible one (group of 17 patients), where the average reduction in LLD was 5.7 mm. After a two-year observation period, these differences were even greater, i.e., by an average of 17.9 mm in the irreversible epiphysiodesis and an average of 12.2 mm in the reversible variant. The author also presented a high revision rate for reversible epiphysiodesis (17.6% to 4.8%), with the main reason being implant migration. He also commented negatively on temporal epiphysiodesis, highlighting its unpredictability for growth cartilage activity after implant removal and possible asymmetries in the shape of the knee joint [21]. All the above-mentioned works indicate that the LLD treatment method using eight-plate implants is not so perfect.

In our analysis, we assessed the impact of eight-plate implants on the width of the knee joint (“TW” parameter). This parameter has not been assessed so far in the available literature. During the treatment with the implants, the width of the tibial base increased compared to the other limb in groups II and III. This parameter increased from 0.9 mm to 5.7 mm (dia. 1.4 mm) in group II (30-month treatment) and from 1.6 mm to 12.2 mm (dia. 7.6 mm) in group III (42-month treatment). This can significantly change the congruence of the knee joint. These results give another argument to opponents of temporal epiphysiodesis who believe that the shorter limb, considered responsible for the occurrence of leg length asymmetry, should be treated for LLD. It is known that changing the surface of the knee joint may accelerate the development of knee arthrosis [22,23,24]. 

In the available literature, authors do not specify the time frame for the use of temporal epiphysiodesis. Most researchers suggest that the so-called safe duration of use of this method should not exceed 24 months. This belief is based on reports provided by the authors of the method, Blount and Clark, on the limitations of temporal epiphysiodesis. They reported that implants kept in the growth cartilage area for longer than 2 years may permanently damage it. However, they do not explain the mechanism by which this damage would occur [25]. These observations were confirmed by Phemister in frequent correspondence with the authors of temporal epiphysiodesis [26]. 

Our research shows changes in the shape of the joint and limb axis when the treatment period is substantially exceeded (24 months). The smallest changes were observed in group I (treatment for up to 18 months). The age of the patients is also important; this group comprised teenagers aged from 12 years and 2 months to 13 years and 10 months (on average, 13 years and 2 months). In other studies comparing epiphysiodeses, the average age of patients ranged from 10 to 14 years at the time of surgical treatment. In 2018, W. Lee presented the results of a comparison of temporal epiphysiodesis, depending on the type of implant used. The group included 19 patients aged from 10.0 to 13.8 years (mean 12.1) [27]. A similar average age of patients treated with epiphysiodesis, i.e., 13.3 years, was presented in 2017 by Bayhan et al. In their study, a larger group of patients was analyzed, i.e., 72 cases with LLD. In the temporal group, an average LLD correction of 12 mm was achieved (41%), and in the irreversible group, of 16 mm after 24 months of treatment [28]. The work covered a treatment period of 24 months, ending with the removal of the implants. In our study, the greatest changes occurred in group III (the youngest patients) and at the longest treatment duration of 48 months.

Limitations of the study: Our work is not without limitations. Firstly, it is a relatively small research group (60 patients), although it is still larger than those in the works of the above-mentioned authors. Moreover, we did not analyze lateral X-rays. This would facilitate the assessment of the shape of the knee joint in the sagittal plane. Despite the use of digital measurement tools, the measurements are not that precise. The use of CT and MRI would facilitate a more accurate assessment of the surface. However, these methods are not used to assess the progress of LLD treatment. It should also be mentioned that the skeletal maturity of the patients was not assessed.

This topic requires further research and the analysis of subsequent groups of patients. The effect of the eight-plate implant on sensitive growth cartilage is still not fully understood. We should look at our actions more broadly and be aware of the consequences they may cause. Thus, there is a need for future long-term follow-up studies to elucidate the potential effects of LLD treatment on the tibial surface.

## Figures and Tables

**Table 1:** Characteristics of participants.

Parameter	Group I	Group II	Group III
Duration of treatment (months)	18	30	42
Number/(boys/girls)	24/(12/12)	24/(18/6)	12/(6/6)
Age y.m average/(range)	13.2/(12.2–13.80)	10.11/(10.2–11.11)	9.0/(8.6–9.6)
Age y.m boys average/(range)	13.0/(12.2–13.11)	11.3/(10.7–11.11)	9.2/(9.0–9.6)
Age y.m girls average/(range)	13.3/(2.11–13.10)	10.6/(10.2–10.11)	8.11/(8.10–9.6)
LLD cm average/(range)	2.0/(1.5–2.8)	2.6/(1.4–3.6)	2.4/(1.6–3.6)
LLD cm boys average/(range)	2.1/(1.5–2.8)	2.6/(1.5–2.8)	2.5/(2.0–3.2)
LLD cm girls average/(range)	2.0/(1.5–2.6)	2.6/(1.4–3.6)	2.4/(1.6–3.6)

y.m—year.months; LLD—leg length discrepancy.

**Table 2:** Changes in the investigated parameters from the beginning to the end of the treatment period.

Parameter	Group I (0–18 Months)			Group II (0–30 Months)			Group III (0–42 Months)		
	Girls	Boys	Total	Girls	Boys	Total	Girls	Boys	Total
Δ D angle (degrees)	6–16 (av. 9.5)	1–15 (av. 8.9)	1–16 (av. 9.1)	8–9 (av. 8.3)	9–15 (av. 12.0)	8–15 (av. 10.2)	8–9 (av. 8.6)	6–14 (av. 10.3)	8–14 (av. 9.4)
Δ α angle (degrees)	1–8.9 (av. 4.9)	5.1–6.8 (av. 7.3)	1–8.9 (av. 5.1)	1.9–2.2 (av. 2.0)	6.3–9.6 (av. 8.3)	1.9–9.6 (av. 5.15)	2.8–7.4 (av. 4.3)	5.7–8.9 (av. 7.3)	2.8–7.4 (av. 5.8)
Δ β angle (degrees)	1.5–16 (av. 6.25)	0.5–2.7 (av. 1.9)	0.5–16 (av. 4)	3.2–3.6 (av. 3.4)	1–7.1 (av. 3)	1–7.1 (av. 3.2)	6.1–8.8 (av. 7.9)	3.9–6.1 (av. 5)	3.9–8.8 (av. 6.45)
Δ MTH (mm)	0.6–2.6 (av. 1.6)	0.7–1.9 (av. 1.4)	0.6–2.6 (av. 1.5)	1.5–2.2 (av. 1.9)	0.5–3.6 (av. 2.3)	0.5–3.6 (av. 2.1)	1.3–2.1 (av. 1.5)	1.2–2.5 (av. 1.9)	1.2–2.5 (av. 1.7)
Δ LTH (mm)	2.4–4.7 (av. 2.4)	0.7–1.7 (av. 1.2)	0.7–4.7 (av. 3.6)	2.6–3.8 (av. 3.4)	0.3–3.8 (av. 2.4)	0.3–3.8 (av. 2.9)	0.3–2.1 (av. 0.9)	2.1–3.6 (av. 2.8)	0.3–3.6 (av. 1.8)
Δ TW (mm)	0.3–17.7 (av. 6.6)	1.8–13.3 (av. 9.4)	0.3–17.7 (av. 8)	0.9–1.1 (av. 1.0)	1.4–5.7 (av. 1.8)	0.9–5.7 (av. 1.4)	1.6–9.9 (av. 4.3)	9.9–12.2 (av. 11)	1.6–12.2 (av. 7.6)

Δ—difference between the measurements at the beginning and after the treatment; D—angle between the medial and lateral lines of the tibial plate inclination; β—angle between the medial line of the tibial plate inclination and the joint line of the tibia; α—angle between the lateral line of the tibial plate inclination and the joint line of the tibia; MTH—dimension measured from the tibial articular line to the highest point in the projection of the medial intercondylar tubercle; LTH—dimension measured from the tibial articular line to the highest point in the projection of the lateral intercondylar tubercle; TW—width of the tibial epiphysis between the farthest points of the proximal tibial epiphysis located on the lateral and medial cortex.

**Table 3:** Statistical significance of the differences (*p*) in values obtained before and after the treatment.

Parameter	G I (0–18 Months)		G II (0–30 Months)		G III (0–42 Months)	
	Girls	Boys	Girls	Boys	Girls	Boys
D angle l	0.0007	0.0102	0.0002	0.0001	0.0039	0.0039
D angle k	0.0004	0.0555	0.2709	0.6358	0.0277	0.5706
α angle l	0.2615	0.4079	0.5492	0.0001	0.4407	0.7419
α angle k	0.0011	0.4936	0.0795	0.6269	0.0609	0.6226
β angle l	0.0117	0.0776	0.6586	0.0007	0.0041	0.0206
β angle k	0.0005	0.6823	0.1617	0.0001	0.1311	0.9943
MTH l	0.0001	0.0001	0.0001	0.0001	0.00001	0.00001
MTH k	0.0103	0.4677	0.0245	0.0001	0.02707	0.2056
LTH l	0.0001	0.0001	0.0001	0.0001	0.0001	0.0001
LTH k	0.7839	0.5299	0.0291	0.0001	0.0001	0.8245
TW l	0.0001	0.0001	0.01231	0.0001	0.0001	0.0001
TW k	0.0001	0.0001	0.0001	0.0001	0.0001	0.0001

l—longer leg; k—shorter leg.

**Table 4:** P probability values (longer leg vs. shorter leg) for the Wilcoxon test—comparison in different time periods for group I.

	Girls						Boys					
Period	D	α	β	MLH	LTH	TW	D	α	β	MLH	LTH	TW
0–6 m	0.0277	0.0281	0.9165	0.0125	0.0047	0.3078	0.4631	0.0022	0.0277	0.0022	0.0995	0.6379
6–12 m	0.0277	0.0121	0.6002	0.0229	0.0229	0.0229	0.2489	0.0022	0.3454	0.6949	0.0281	0.1579
12–18 m	0.0277	0.0121	0.4631	0.0022	0.0995	0.3078	0.0277	0.1579	0.6002	0.0229	0.0121	0.6379

D—decrease in Δ D angle; α—increase in Δ α angle; β—increase in Δ β angle; MLH—increase in medial tubercle height; LTH—increase in Δ medial tubercle height; TW—increase in Δ width of the tibial epiphysis.

**Table 5:** P probability values (longer leg vs. shorter leg) for the Wilcoxon test—comparison in different time periods for group II.

	Girls						Boys					
Period	D	α	β	MLH	LTH	TW	D	α	β	MLH	LTH	TW
0–6 m	0.0678	0.1159	0.0277	0.9165	0.9165	0.0277	0.0707	0.0011	0.0096	0.0479	0.0002	0.0011
6–12 m	0.0277	0.9165	0.0479	0.0277	0.0277	0.0277	0.0002	0.0096	0.0033	0.0018	0.7174	0.0151
12–18 m	0.0277	0.0277	0.9165	0.0277	0.1158	0.9165	0.0011	0.6791	0.4204	0.0311	0.0014	0.4379
18–24 m	0.0277	0.0277	0.1158	0.1797	0.0277	0.0277	0.2667	0.7771	0.0006	0.0049	0.0582	0.4459
24–30 m	0.4652	0.0277	0.4631	0.0277	0.0277	0.0277	0.4459	0.0096	0.0057	0.0386	0.0084	0.0311

D—decrease in Δ D angle; α—increase in Δ α angle; β—increase in Δ β angle; MLH—increase in medial tubercle height; LTH—increase in Δ medial tubercle height; TW—increase in Δ width of the tibial epiphysis.

**Table 6:** P probability values (longer leg vs. shorter leg) for the Wilcoxon test—comparison in different time periods for group III.

	Girls						Boys					
Period	D	α	β	MLH	LTH	TW	D	α	β	MLH	LTH	TW
0–6 m	0.1158	0.1158	0.0277	0.1158	0.0277	0.0277	0.1158	0.1158	0.9165	0.4631	0.4631	0.1158
6–12 m	0.0678	0.0277	0.9165	0.0277	0.0277	0.0277	0.0678	0.9165	0.9165	0.4631	0.0277	0.9158
12–18 m	0.0678	0.0277	0.0277	0.4631	0.9165	0.1158	0.0678	0.4631	0.0277	0.4631	0.4631	0.1158
18–24 m	0.4652	0.0277	0.0277	0.0277	0.0277	0.9165	0.0678	0.9165	0.9165	0.0277	0.0277	0.1158
24–30 m	0.0277	0.1158	0.0277	0.0277	0.0277	0.0277	0.1797	0.9165	0.9165	0.9165	0.4631	0.4631
30–36 m	0.9165	0.9165	0.0277	0.0277	0.0277	0.0277	0.0277	0.9165	0.0277	0.4631	0.4631	0.1158
36–42 m	0.1158	0.9165	0.0277	0.1158	0.1158	0.0277	0.1158	0.0277	0.9165	0.4652	0.1158	0.1277

D—decrease in Δ D angle; α—increase in Δ α angle; β—increase in Δ β angle; MLH—increase in medial tubercle height; LTH—increase in Δ medial tubercle height; TW—increase in Δ width of the tibial epiphysis.

## Data Availability

The data presented in this study are available upon request from the corresponding author.
